# Vibration Reduction and Explosion Control Investigation for an Ultra-Shallow Buried Tunnel under Crossing Buildings Based on HHT Analysis

**DOI:** 10.3390/s23177589

**Published:** 2023-09-01

**Authors:** Rui Xu, Jichun Zhang, Bian Wu, Feng-Liang Zhang

**Affiliations:** 1 Tianjin Municipal Engineering Design & Research Institute, Tianjin 300392, China; azuremoonxr@my.swjtu.edu.cn; 2 School of Civil Engineering, Southwest Jiaotong University, Chengdu 610031, China; jczhang2012@swjtu.edu.cn; 3 School of Civil and Environmental Engineering, Harbin Institute of Technology, Shenzhen 518055, China; 20b954033@stu.hit.edu.cn; 4 Guangdong Provincial Key Laboratory of Intelligent and Resilient Structures for Civil Engineering, Harbin Institute of Technology, Shenzhen 518055, China

**Keywords:** HHT, ultra-shallow buried tunnel, cutting hole, instantaneous energy, vibration velocity, millisecond delay blasting, wave interference vibration reduction

## Abstract

With the rapid development of underground space utilization, the excavation of new tunnels with ultra-shallow under crossing buildings using the drilling and blasting method is gradually increasing. The blasting vibration will undoubtedly affect the surrounding buildings. Reducing the impact of blasting vibration on ground buildings has become an important technical challenge faced by tunnel blasting technicians. The inlet end of the Xi’an-Chengdu High-Speed Railway Xiannvyan Tunnel passes below a village through an ultra-shallow buried section; as a result, blasting vibration control is a major concern. A design scheme for a 0.6 m footage in tunnel was proposed and verified through field tests. A 0.8 m footage scheme and 1.8 m footage millisecond interference vibration reduction scheme were proposed, respectively. Based on the HHT analysis, by comparing the surface vibration velocities and instantaneous energy obtained from the millisecond delay detonation of cutting holes and the detonation of different charging schemes, we found that the free surface, mass of single dynamite charges, and tunnel burial depth had significant influences on the surface vibration.

## 1. Introduction

Blasting vibration control is an important topic in tunnel construction. Nowadays, more and more tunnels are being constructed, and the tunnel section becoming larger in size. As an effective means of analyzing vibration characteristics, the tunnel blasting vibration signal analysis method has been widely used. A blasting vibration wave is an unstable signal, its frequency composition is complex and changeable with one or several main frequency components, and certain frequency components could have an impact on structures [1,2,3]. In 1998, Norden E Huang, a Chinese−American from NASA, proposed the Hilbert−Huang Transform (HHT). It was considered a significant breakthrough in linear steady-state spectral analysis based on Fourier transform [4]. It has many advantages for analyzing nonlinear non-stationary signals, such as completely overcoming the constraints of linearity and steady-state, having stronger local characteristics, and reflecting the true physical meaning of the signal. Wu et al. obtained the energy curve using the HHT method to analyze the blasting time of the new free surface, so as to improve the blasting parameters [5]. Ma et al. analyzed the influence of blasting on the adjacent tunnel using HHT analysis [6]. Song and Hou et al. analyzed the damage to the surrounding buildings from the perspective of blasting energy, which could supplement and improve the existing safety criteria, but a lot of experimental and engineering practice data are still needed [7,8].

In order to control the tunnel blast, measures such as shortening the individual cycle of excavation, opening up the exterior in stages, adjusting the hole sizes incrementally, and reducing the maximum section charge of dynamite used are often adopted [9,10,11]. In recent years, research into controlled blasting has made fruitful progress [11,12,13,14].

Engineering blasting uses mostly differential blasting as the main method for vibration reduction. Interference vibration reduction uses differential blasting technology to arrange explosive charges into groups, which are then detonated successively according to specific time intervals [12,15,16]. Segment medicine packets form segmented vibrations and segmented delay forms a certain phase difference. These segmented vibrations interfere and superimpose at the appropriate moment, so that the vibration amplitude after interference is reduced, in order to achieve the purpose of vibration reduction. The delay time control accuracy of ordinary non-electric detonators is extremely poor, and the delay error range is 10–120 ms, which cannot achieve the purpose of ideal interference vibration reduction. In the late 1980s, the Australian ICI Company developed high-precision detonators that could freely adjust the delay time, and found that a testing interval of 25 ms produced the best damping effect [17]. Zhang et al. proposed the superposition theory of seismic wave phase difference waveform, which can effectively reduce the vibration velocity [18]. Iwano et al. proposed a single blasting waveform superposition method that can be used to obtain the optimal millisecond time [19]. According to the research results of the Changsha Institute of Mining and Metallurgy, the reasonable interval time for most ore rocks is between 8.4–12.7 ms [20]. Because the electric detonator has not been fully popularized and the interference delay time needs to be accurately designed, interference reduction has remained in the theoretical or experimental stages.

In order to reduce the surrounding influence, the construction of the shallow-buried large-span tunnel mostly adopts the scheme of reducing single-cycle footage. The study of interference shock reduction may solve the problem of small single-cycle footage. However, the research on interference blasting reduction is still in the theoretical stages, and most of it is still based on the existing safety requirements needed to meet the conditions at a certain frequency. The influence of shallow-buried tunnel blasting on the surrounding area should not only consider the blasting velocity, but also the characteristics of high-frequency tunnel surface vibration and large output energy. The research content of this paper was based on the Xiannvyan Tunnel Project of the Xi’an-Chengdu Passenger Dedicated Line. The entrance mileage of this tunnel at the third bid of Xi’an-Chengdu Passenger Dedicated Line passes underneath a residential building. The blasting vibration needs to be controlled and the required vibration speed needs to be less than 2.0 cm/s. According to previous vibration monitoring results, the vibration above the tunnel face seriously exceeded the design requirements. Therefore, when tunneling underneath residential buildings, it is necessary to optimize the previous blasting plans. In this paper, the blasting vibration reduction scheme mainly focused on reducing the charge quantity of explosives and the application of waveform interference for vibration reduction.

Blasting vibration monitoring at the entrance of the Xiannvyan Tunnel was carried out for several months. Based on the large amount of monitoring data, the law of blasting vibration was obtained using the HHT unstable wave analysis method. The instantaneous energy spectrum obtained from HHT was used as the main means to analyze the propagation law of the blasting energy. Through the tunnel blasting construction design of different dynamite quantities, different dynamite structures, and blasting methods, the best blasting vibration reduction scheme was obtained by comparing the various schemes from two aspects—vibration speed and instantaneous energy.

## 2. Target Tunnel

**Figure 1:**
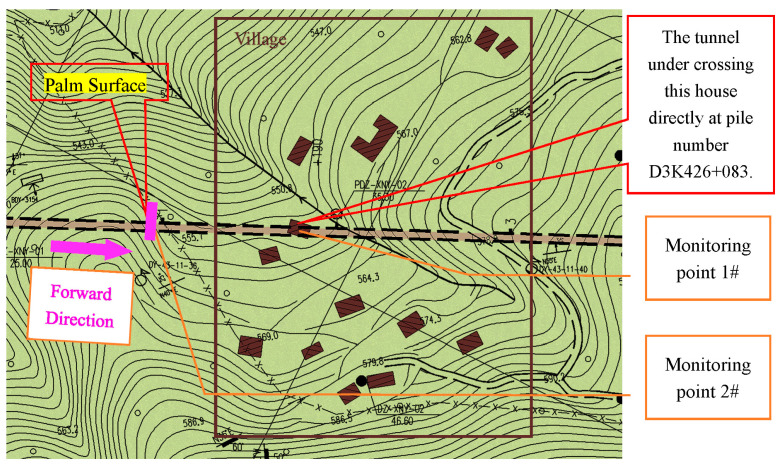
Planar relation between the village and Xiannvyan Tunnel.

The Xiannvyan Tunnel is located in Jiange County, Guangyuan City, China. The tunnel entrance pile number is D3K425+953, and it crosses under a village at D3K426+090~D3K426+260. The length of the underpass is 170 m, and the buried depth of this section is 15 m~25 m and it is part of the ultra-shallow buried section. Considering the village building structure, the blasting vibration needs to be less than 2.0 cm/s. The planar relation between the village and Xiannvyan Tunnel is shown in Figure 1.

### 2.1. Blasting Scheme for Tunnel Construction in Non-Vibration Reduction Section

**Figure 2:**
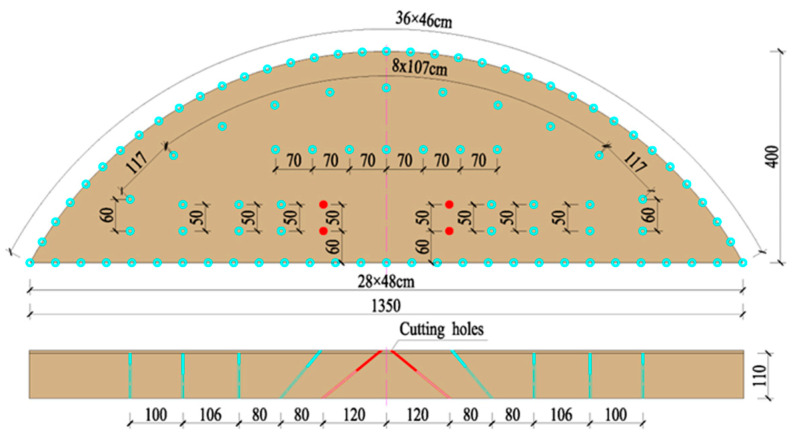
Arrangement of holes (cm).

The tunnel was excavated using a three-step excavation method. The single-cycle footage of the upper step was 1 m; the excavation width was 13.5 m, the excavation height was 4 m, and the volume of single-cycle blasting rock was 38.2 m^3^. The angle between the cutting holes and the pale surface was 45°. The depth of the hole was 1.2 m and the distance of the distance was 0.5 m. The arrangement of holes on the palm surface is shown in Figure 2, and the blasting parameters are shown in Table 1.

### 2.2. Blasting Vibration Monitoring Method

#### 2.2.1. Test Instrument

The vibration data were collected using a T4850 blasting vibration detector. The sampling frequency was set to 8K and the recording time was 2 s.

#### 2.2.2. Detector Layout

The point #1 monitoring was arranged next to the target building. Another monitoring point (#2) was arranged above the palm surface, and it moved along in the direction of the tunneling. The position and arrangement of the monitoring points are shown in Figure 1.

#### 2.2.3. Monitoring Results

Because the middle and the lower layers benefited from the free surface effect, their vibration velocity was small, and so vibration monitoring was mainly carried out on the upper layer. The monitoring lasted for more than two months and more than 30 groups of data were collected. Table 2 shows the signals monitored during 3 events. The monitoring event 1 include a 2 m long footage blasting waveform as a field test (six cutting holes were arranged with 0.7 kg of charge per hole).

**Figure 3:**
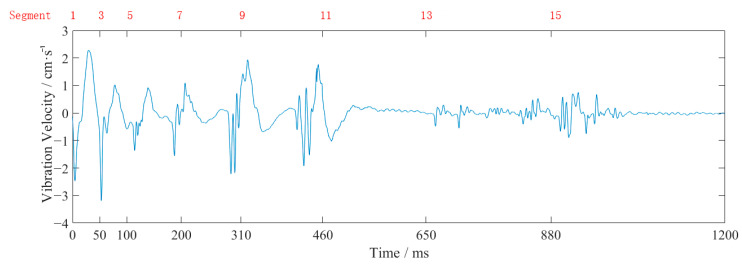
The vertical waveform at monitoring point #2 of event 2.

Figure 3 shows the vertical waveform of monitoring point #2 for event 2. From Table 2 and Figure 3, it can be seen that the closer to the source, the larger the vibration frequency and velocity. The vibration velocity had multiple peaks, which coincided with the use of differential blasting. The vibration time lasted about 1 s, and the vibration velocity generated by the blasting of the cutting holes was the largest. The vibration velocity of this explosion above the pale surface was far larger than the design requirement of 2 cm/s.

## 3. Blasting Vibration Analysis Based on HHT 

### 3.1. Implementation of HHT Time−Frequency Analysis Method

The HHT analysis method mainly included the Empirical Mode Decomposition (EMD) and Hilbert−Huang Transform (HHT), and the main process is as follows:

1. The upper envelope $X_{max}\left(t\right)$ and lower envelope $X_{min}\left(t\right)$ of the original signal $X\left(t\right)$ are obtained based on the local extreme value point. Subtract the mean $m\left(t\right)$ of the upper and lower envelopes from the original sequence $X\left(t\right)$ and obtain $c\left(t\right)$. If it satisfies the IMF condition, the single ($c\left(t\right)$) is stored as the first IMF component imf(t), otherwise the original single is replaced with $c\left(t\right)$ and the above process is repeated until the IMF component appears.
2. The IMF component is removed from the original signal, and the residual signal is further analyzed to find the next IMF component, which becomes the new primary signal. This occurs until the remaining sequence $r\left(t\right)$ becomes a monotonic function or is less than a certain value.
3. For each IMF component, Hilbert transform is performed:
  (1) $$H\left[imf\left(t\right)\right]=\frac{1}{\pi }PV\int_{-\infty }^{+\infty }\frac{imf(t^{\prime })}{t-t^{\prime }}dt^{\prime },$$
  where PV represents the Cauchy principal value.

A three-dimensional signal space can be created using time, instantaneous frequency, and signal amplitude, which can be expressed as contours on the time and instantaneous frequency plane. This three-dimensional signal represents the amplitude distribution as the Hilbert spectrum, and the signal expression is shown as follows:

(2) $$H(\omega ,t)=\sum_{i=1}^{n}a_{i}(t)e^{\int \omega_{i}\left(t\right)dt},$$

where $a\left(t\right)$ is the amplitude function:

(3) $$a\left(t\right)=\sqrt{{imf}^{2}\left(t\right)+H^{2}[imf\left(t\right)]},$$

and θ(t) is the instantaneous frequency function:

(4) $$\omega \left(t\right)=\frac{d\theta (t)}{dt},$$

(5) $$\theta \left(t\right)=\arctan\left(\frac{H\left[imf\left(t\right)\right]}{imf\left(t\right)}\right).$$

The Hilbert marginal spectrum H(ω,t) can be obtained by integrating the time obtained from the following equation:

(6) $$H(\omega )=\int_{0}^{T}H\left(\omega ,t\right)dt.$$

The instantaneous energy spectrum is shown as follows, and it can be seen as the instantaneous change in energy:

(7) $$IE(t)=\int_{\omega }H^{2}\left(\omega ,t\right)d\omega .$$

### 3.2. HHT of Bursting Vibration Signals

**Figure 4:**
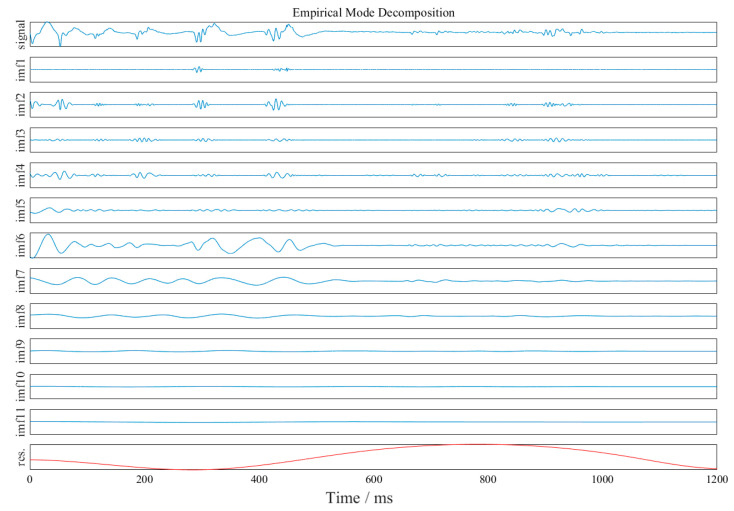
EMD waveform. (The blue lines are the IMF components, i.e., imf_1_-imf_11_ and the red line is residual component, i.e., res.)

**Figure 5:**
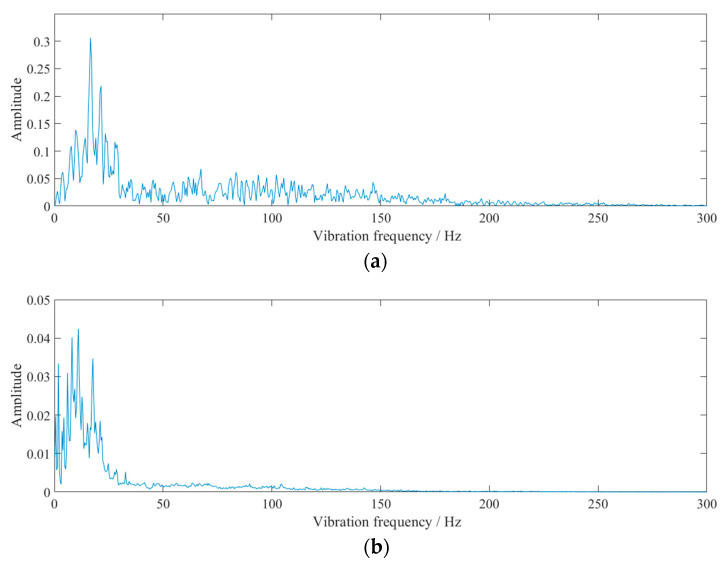
Comparison between the frequency spectrum and the marginal spectrum: (**a**) frequency spectrum and (**b**) marginal spectrum.

**Figure 6:**
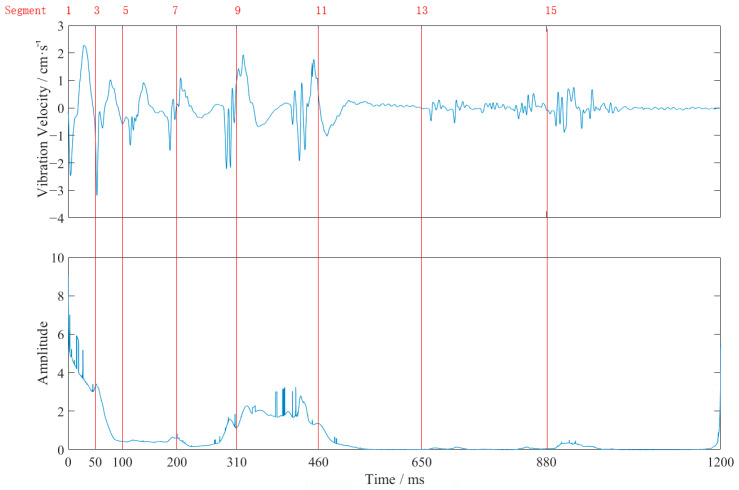
Comparison between the original signal and the instantaneous energy.

The waveforms at point #2, in event 2 (Table 2 and Figure 3), were analyzed as an example. The obtained EMD waveform is shown in Figure 4. The marginal spectrum of the original signal was obtained using Equation (6), and the comparison with the frequency spectrum (obtained by Fourier transform) is shown in Figure 5. The instantaneous energy spectrum was obtained using Equation (7) and the comparison with the original signal is shown in Figure 6.

The original signal was decomposed into 11 IMF components and 1 residual component. The decomposed waveform frequencies were arranged as imf_1_–imf_11_ and res, from high to low.

The marginal spectrum and frequency spectrum were both mainly concentrated in 0~50 Hz. When applying Fourier transform with the same amplitude across any frequency, false frequencies may emerge in order to fit the original signal. Thus, the frequency of 50~150 Hz in Figure 5a is clearly visible and Fourier transform is not suitable for processing non-stationary signals. The Hilbert transform reflects the relationship between time and frequency. A frequency may exist, but the existence time may be very short. In addition, the marginal spectrum can effectively represent the energy per unit frequency. The results of HHT conform to the original waveform law, which reflects the practicability of HHT. Because the natural frequencies of the buildings were low and mostly in the range of 10 Hz, the relatively low vibration frequency could potentially have a significant adverse impact on the buildings.

The energy accumulation in a certain frequency section can be seen from the margin spectrum. It can be observed that the energy was mainly distributed in the frequency range of 0~50 Hz. However, the marginal spectrum could not reflect the energy time characteristics; this could be analyzed by combining the instantaneous energy spectrum with the marginal spectrum.

It can be seen from Figure 6 that the maximum vibration velocity did not occur at the same time as the maximum instantaneous energy; the latter occurred during the first segment at the moment of the blasting cartridge. After the detonation of the third segment’s dynamite, the vibration velocity increased instantaneously to the maximum. However, the instantaneous energy did not reach the maximum because the vibration frequency was higher here.

Instantaneous energy can reflect the instantaneous input energy of structures, and the energy represented consistently over time can represent the continuous input of energy. If the instantaneous input energy of the building is greater than its critical damage energy value, it will cause large deformations or even lead to destruction. Even if the damage threshold is not reached, if the fatigue damage threshold of the building is reached, there will be a cumulative damage effect on the building. Therefore, the blasting vibrations consisting of spikes may not cause obvious damage to a building after several blasts, but if the peak energy amplitude exceeds the fatigue failure value, it will still cause damage to the structure as a result of accumulated fatigue.

## 4. Simulation and Analysis of Blasting Vibration Reduction

According to the field geology and the spatial relationship of the tunnel surface, a finite element model (FEM) was established by ANSYS. The blasting simulation was calculated by LS-DYNA, and the waveforms for the monitoring point were extracted using LS-PROPOST.

### 4.1. Development of FEM

#### 4.1.1. Modeling and Meshing

According to the spatial layout of the test monitoring point of event 1, a finite element model was built. The length of the model along the tunnel excavation direction was set as 5.0 m, the tunnel buried depth (from the arch crown to the surface) was 11.7 m, and the model size was 60.0 m × 42.7 m × 5.0 m. The excavation width of the upper step of the tunnel was about 13.5 m, the distance from the bottom of the tunnel to the model boundary was 18.1 m, the distance from the top of the tunnel to the upper boundary was 11.7 m (with rock layer of 7.7 m, and soil layer of 4.0 m), and the distance from the left to the right boundary was 22.2 m. As only the cutting blasting vibration velocity was controlled, only the cutting hole was established in the model. The cutting hole was simplified in the vertical direction with a depth of 2.0 m. The dynamite was 60 cm from the bottom of the upper layer of the excavation; the height difference of the three pairs of cutting holes was 50 cm, and each hole was 30 cm from the tunnel center line. The total charge of dynamite in the hole was 4.2 kg, with 0.7 kg for each hole; the length of the charging structure was 70 cm and the hole was plugged using 130 cm.

**Figure 7:**
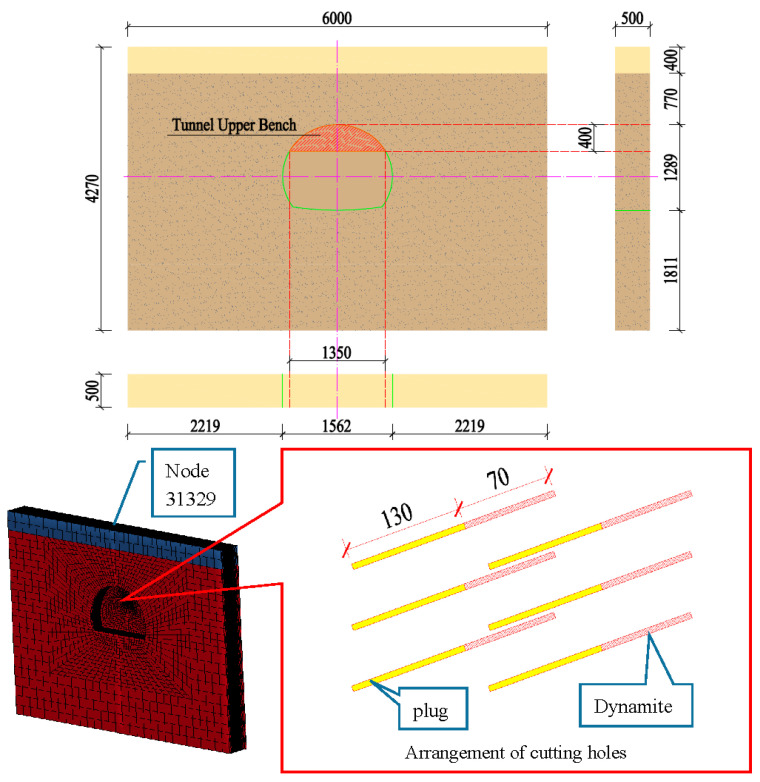
Finite element meshing model (cm).

The model dynamite, geotechnical bodies, and plugging sections all adopted SOLID164 solid units, and the maximum unit size was controlled at 150 cm. The dynamite size was controlled at 10 cm. The total number of elements in the whole model was 133,032. The upper boundary of the model and the tunnel excavation section were the free boundaries. The rock and soil boundaries were vertical constraints, and non-reflective boundary conditions were applied. The model size and the finite element meshing model are shown in Figure 7.

#### 4.1.2. Parameter Selection of FEM

The simulation of the rock adopted the dynamic plastic kinematic material model (*MAT_PLASTIC_KINEMATIC), the upper soil layer adopted the ideal elastoplastic model (*MAT_DRUCKER_PRAGER), and the dynamite adopted high-energy dynamite material (*MAT_HIGH_DYNAMITE_BURN, equation of state *EOS_JWL). The Lagrange grid was used for meshing, and the dynamite rock mass adopted shared nodes. The values of the key parameters are shown in Table 3, Table 4 and Table 5.

#### 4.1.3. Model Validation

**Figure 8:**
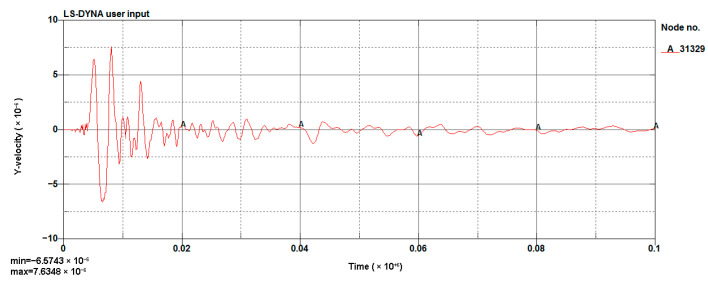
Cutting hole blasting waveform of the model (g-cm-µs).

**Figure 9:**
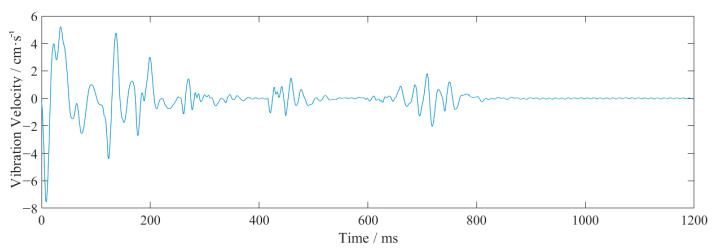
Cutting hole blasting waveform of monitoring point #2.

The LS-DYNA calculation of the model was performed, and the vibration velocity of point 31329, which was located directly above the palm surface, is shown in Figure 8. The maximum vibration velocity was 7.63 cm/s, at an approximate time of 0.008 s. Contrasting the test monitoring point waveform (the signal monitored #2 at event 1, Table 2), the maximum vibration velocity of the original signal (see Figure 9) was 7.55 cm/s, and the maximum vibration velocity time was around 0.006 s. The maximum vibration velocity generation time was different, because the monitoring data for the real and model trigger times were different. The model started timing at the time of detonation, while the actual instrument had a vibration trigger that occurred before timing. From the simulation results, it can be seen that the model reflected the relationship between the charge and the vibration velocity well and simulated the vibration of monitoring point #2 effectively.

### 4.2. Reducing Vibration by Shortening Footage 

#### 4.2.1. Reduced Single-Cycle Footage Scheme Design

**Figure 10:**
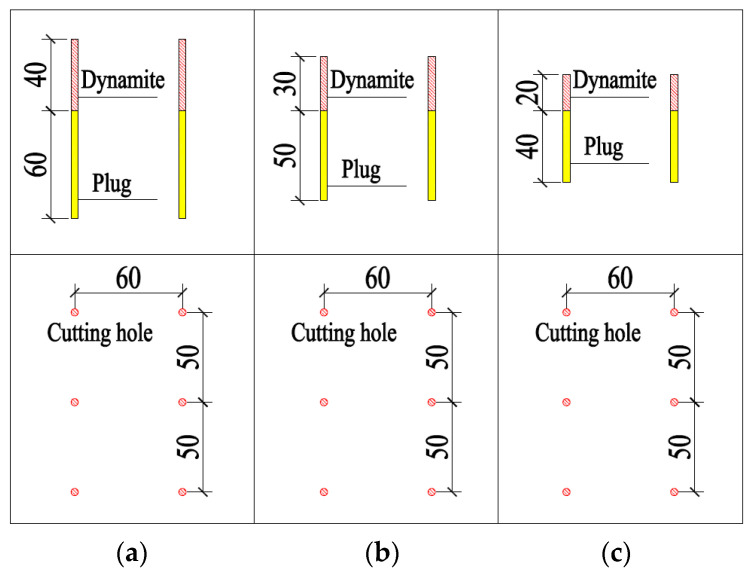
Cutting holes scheme (cm): (**a**) for the 1 m section, the charge is 2.4 kg; (**b**) for the 0.8 m section, the charge is 1.8 kg; (**c**) for the 0.6 m section, the charge is 1.2 kg.

Based on the previous analysis, shortening the single-cycle footage and reducing the single blasting dynamite charge were the main methods for reducing the blasting vibrations. From the signal obtained using the test monitoring point (the signal monitored #2 at event 1; Table 2), for a 2 m section with the charge in blasting dynamite reaching 4.2 kg, the blasting vibration velocity reached 7.55 cm/s, which was much greater than the design requirements. By shortening the footage to reduce the charge of dynamite, the design was carried out on 1 m, 0.8 m, and 0.6 m sections, respectively. The design is shown in Figure 10 and the corresponding finite element models were established for the analysis.

#### 4.2.2. Simulation Results and Analysis

**Figure 11:**
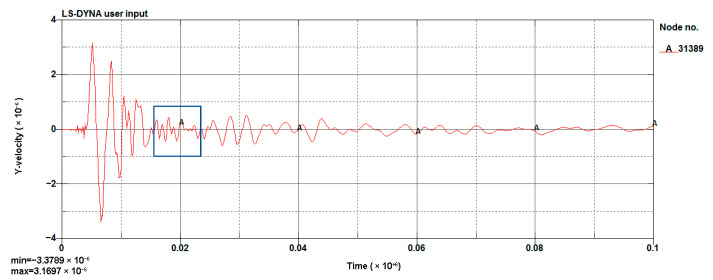
Simulated waveform of a 1 m section (g-cm-µs).

**Figure 12:**
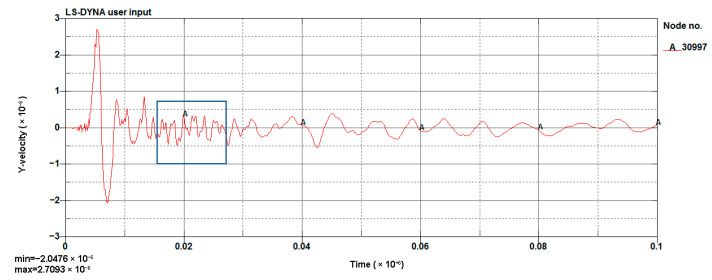
Simulated waveform of a 0.8 m section (g-cm-µs).

Figure 11 shows the simulation of a blasting waveform of a 1 m section above the palm surface. The vibration velocity of the waveform was 3.379 cm/s. Figure 12 shows the simulation of a blasting waveform of a 0.8 m section. The vibration velocity of this waveform was 2.709 cm/s. The simulation results were greater than the design requirements, and this scheme could not be applied in an engineering context.

**Figure 13:**
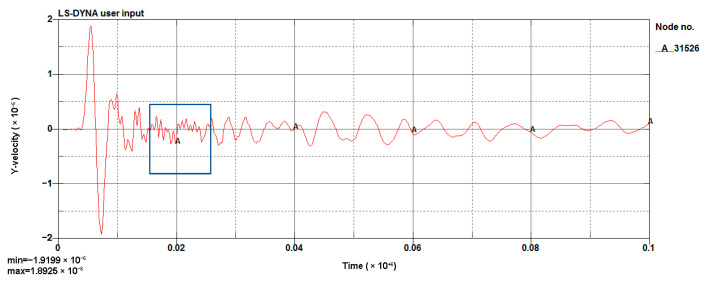
Simulated waveform of a 0.6 m section (g-cm-µs).

Figure 13 shows the simulation of a blasting waveform of a 0.6 m section. The vibration velocity was 1.919 cm/s and the simulation results met the design requirements. Therefore, this scheme was used for field testing.

The amount of blasting charge had a significant impact on the blasting intensity; the charge of explosives decreased from 2.4 kg to 1.2 kg and the vibration velocity decreased from 3.379 cm/s to 1.919 cm/s. The relationship between vibration velocity, distance of the monitoring points, and the single segment detonation quantity was fitted using Sadov’s formula:

(8) v=KQ13Ra,

where v is the vibration velocity, K is the coefficient related to the blasting conditions, Q is single segment detonation quantity, R is the distance of monitoring points, and a is the vibration attenuation coefficient. 

After calculating the logarithm on both sides of Formula (8), it can be transformed into the following:

(9) $$\ln v=\frac{a}{3}\ln Q+(\ln K-a\ln R).$$

**Figure 14:**
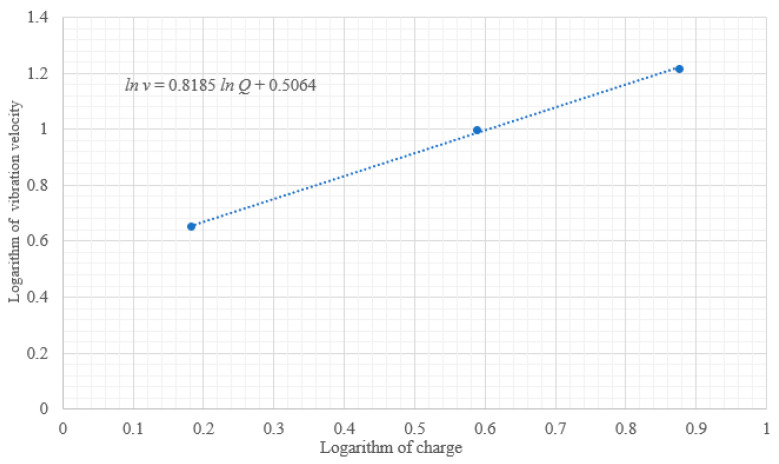
The relationship between the charge of dynamite and the vibration velocity.

Using curve fitting, the relationship between the charge of explosives and the vibration velocity is shown in Figure 14, where a linear relationship can be observed. The natural indices on both sides of the formula in Figure 14 were calculated using ln⁡v=0.8185ln⁡Q+0.5064. Under the same geological conditions, when the charge depth was about 11.8 m, the vibration charge of dynamite formula was as follows:

(10) $$v=1.6593Q^{0.8185}.$$

**Figure 15:**
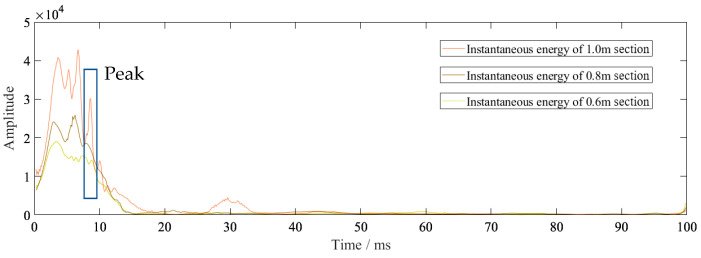
Instantaneous energy spectrum of 1 m, 0.8 m, and 0.6 m sections.

Figure 15 shows the instantaneous energy spectrum for 1 m, 0.8 m, and 0.6 m sections. It can be seen that the 1 m section produced a peak after the main energy and the 0.8 m section also had a similar result. This was due to the reflection of the layer. From the vibration velocity figures (Figure 11, Figure 12 and Figure 13), it can be seen that the vibration velocity entered a very small and unstable frequency blasting vibration (marked in the blue box) and then entered a relatively stable vibration. When reaching a relatively stable signal, we considered the blasting effect to be over. The very small and unstable frequency blasting vibration was partly a result of the main blasting vibration. It was also due to the reflection of the layer; the reflected waves resulted in mutual interference. Corresponding to the time in Figure 15, the instantaneous energy was very small.

### 4.3. Field Test of 0.6 m Footage

#### 4.3.1. Field Test Description

**Figure 16:**
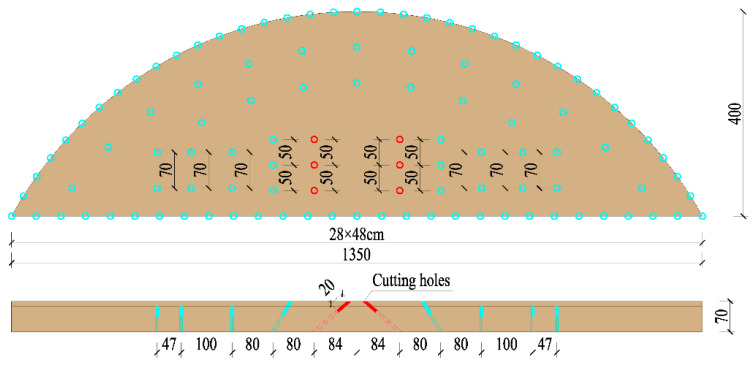
Arrangement of cutting holes (cm).

In order to verify the feasibility of the 0.6 m section, a field blasting test was carried out. The test was carried out at D3K426+070 (event 2). The source depth was 11.8 m. Figure 16 shows the design of the charge structure for the upper layer, and Table 6 shows the charging parameters of the cutting holes. Once again, monitoring point #1 was located next to the target building and point #2 was above the palm surface.

#### 4.3.2. Test Vibration Results and Analysis

**Figure 17:**
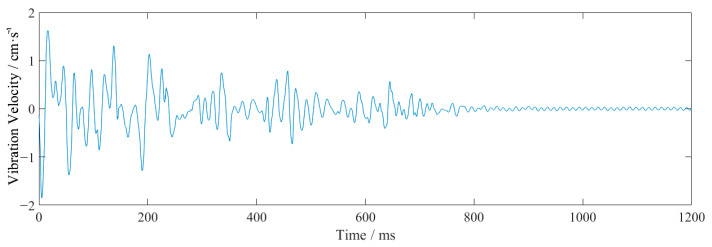
The waveform in the vertical direction for monitoring point #2.

**Figure 18:**
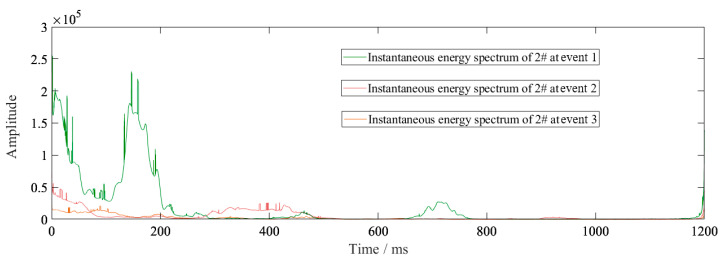
Instantaneous energy spectrum of monitoring point #2 of event 1, event 2, and event 3.

The waveform in the vertical direction for monitoring point #2 is shown in Figure 17 and the field blasting monitoring data in the vertical direction are shown in Table 7. Table 8 shows a comparison of the field blasting data and simulation data. Figure 18 shows the instantaneous energy spectrum comparison of a single charge monitored during event 1, event 2 and event 3 (Table 8) at point #2. 

From Figure 17 and Table 7, it can be seen that the vibration velocity of the field test was 1.852 cm/s, which was close to the simulation result of 1.919 cm/s, which met the design requirements of 2.0 cm/s. This verifies that this design could be applied when crossing under the village.

As mentioned previously, the field blasting data of event 3 were relatively consistent with the simulation data, showing that the model was valid. The cutting hole charge of event 1 was larger than that of event 3, and it could be seen that the vibration velocity was significantly reduced. It can be generally verified that the energy is proportional to the square of velocity, which can be denoted as follows:

(11) $$IE=av^{2}+E\left(f\right),$$

where $E\left(f\right)$ is the function of energy related to frequency. 

**Figure 19:**
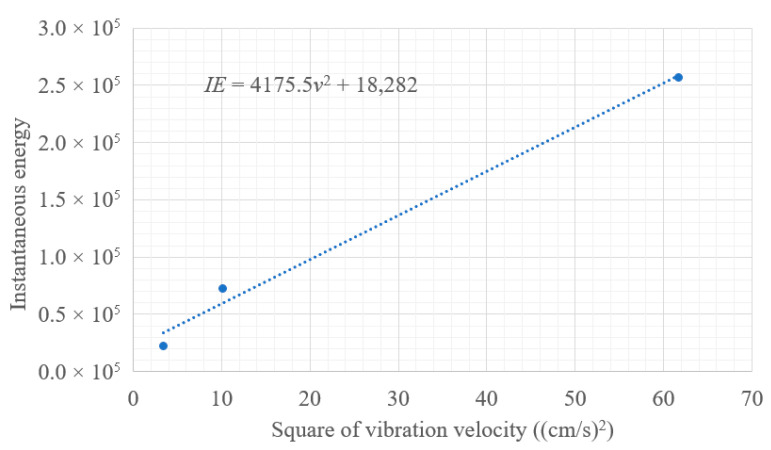
The relationship between vibration velocity and instantaneous energy.

The relationship between vibration velocity and instantaneous energy are shown in Figure 19. The value of $E\left(f\right)$ was 18,282. It can be seen from the figure that the square of velocity and instantaneous energy was a linear relationship. Because the blasting frequency range was mainly between 0~50 Hz, the energy related to frequency was invariant. It can be considered that, regardless of the magnitude of the vibration, the energy reflected by the frequency under the same geological conditions should be close to a fixed value. In this blasting project, the instantaneous energy value of 1.2 kg charge was 22,350 and it contained frequency energy of 18,282. The smaller the vibration velocity, the greater the proportion of energy reflected by frequency.

## 5. Improved Scheme of Blasting

In the previous section, three kinds of tunnel blasting schemes with different length sections were proposed. However, for the three simulated schemes, only the 0.6 m section met the design requirements. Tunnel D3K426+090~D3K426+260 crosses under the village area and its length is 170 m. If the single-cycle section was 0.6 m, it would affect the construction. Therefore, the other two schemes will be improved on in this section.

### 5.1. Improved Scheme of 0.8 m Footage

As the single-segment blasting charge was the main factor of the vibration, a millisecond delay blasting technique was used to cut the holes. In this case, two schemes were designed to reduce the surface vibration velocity. 

**Figure 20:**
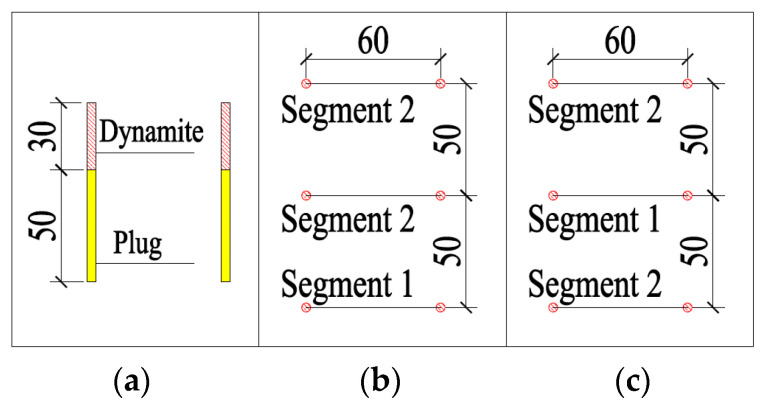
Optimal design of a 0.8 m section of tunnel (cm): (**a**) the charging length diagram. (**b**) Scheme A: the lower pairs of holes are detonated by the first segment detonator (0 ms), and the middle and upper are detonated by the second segment detonator (25 ms). (**c**) Scheme B: the middle pairs of holes are detonated by the first segment detonator (0 ms) and the lower and upper are detonated by the second segment detonator (25 ms).

**Figure 21:**
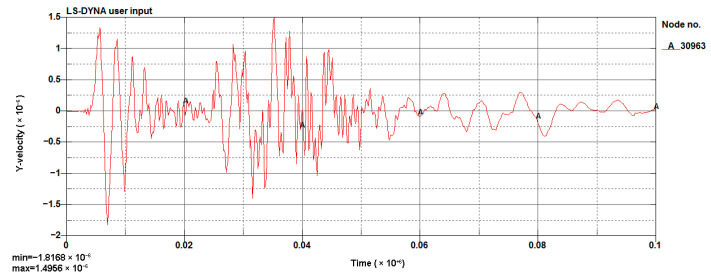
The waveform of scheme A (g-cm-µs).

**Figure 22:**
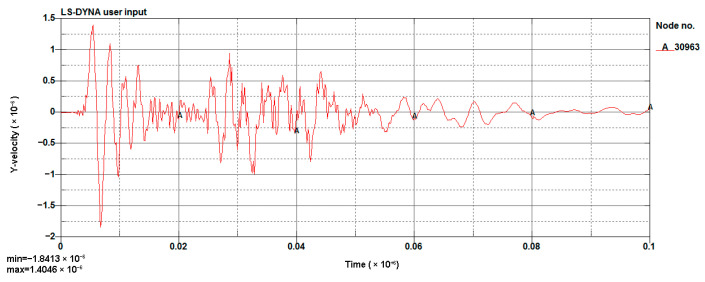
The waveform of scheme B (g-cm-µs).

The scheme design diagram is shown in Figure 20, and the model results are shown in Figure 21 and Figure 22.

This time, node 30963 (this point is located above the palm face) was taken as the monitoring point. The maximum vibration velocity was reduced to 1.8 cm/s (the result for scheme A is 1.8168 cm/s and for B is 1.8463 cm/s) from 2.709 cm/s (the result of 0.8 m footage in Section 4.2), and the vibration detonation by the second segment detonator was far less than the vibration caused by the first segment.

**Figure 23:**
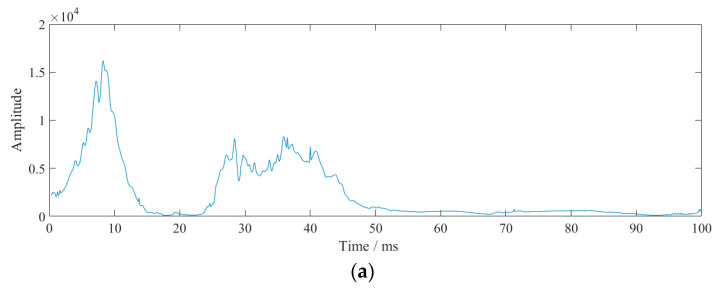

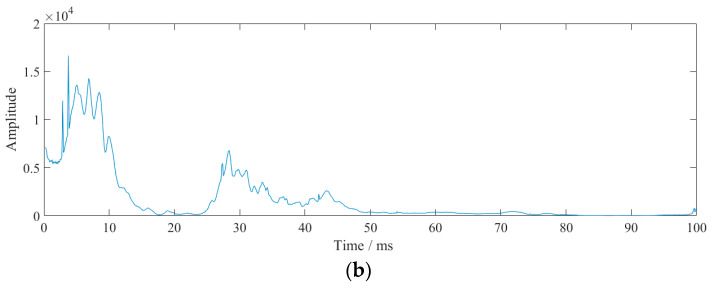
Instantaneous energy composition between (**a**) the instantaneous energy spectrum of scheme A and (**b**) the instantaneous energy spectrum of scheme B.

It was found that the first segment blasting retained a significant vibration advantage, even though the charge of dynamite in the second segment was twice that of the first segment. The simultaneous vibration velocity of the middle and upper cutting holes that were simultaneously detonated was greater than that of the upper and lower cutting holes that were simultaneously detonated. Figure 23 shows the instantaneous energy spectrum of the two designs obtained using HHT. From these figures, it was seen that the resulting instantaneous frequency spectra were also very similar in shape, with two energy peaks appearing at almost the same time in both figures. The instantaneous energy of the detonation of the second segment in scheme A was large, and it was about twice that of the initiating dynamite pack in scheme B. This was because the upper and lower cutting holes all had free surfaces in scheme B. A lot of energy was released from the free surface during blasting. Compared with the two schemes, the vibration velocity obtained by scheme A was almost the same as that of method B, but the total energy was much larger than that of method B.

The 0.8 m section met the design requirements. Considering that the delay error of the ordinary detonator was 10–120 ms and the segment was limited, this scheme could still be tested, but the deviation was relatively large, and it was not easy to apply to field construction.

### 5.2. Improved Scheme by Wave Interference Vibration Reduction

**Figure 24:**
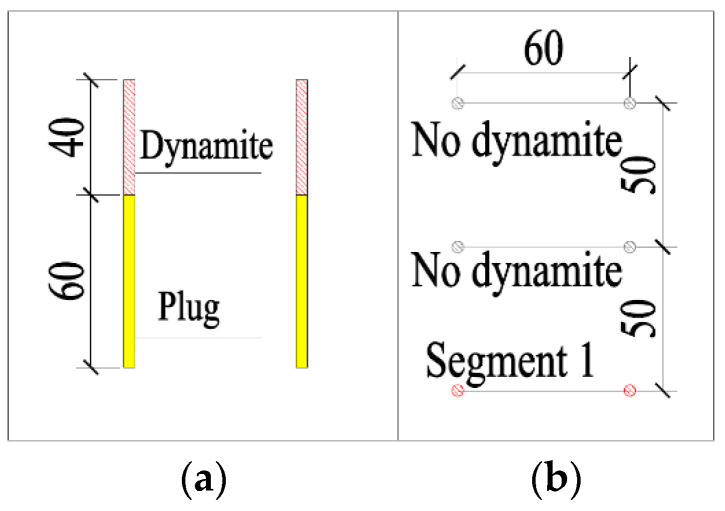
The results of blasting for the lower pairs of holes for a 1m section (cm): (**a**) the charging length diagram; (**b**) the segment arrangement of detonators.

**Figure 25:**
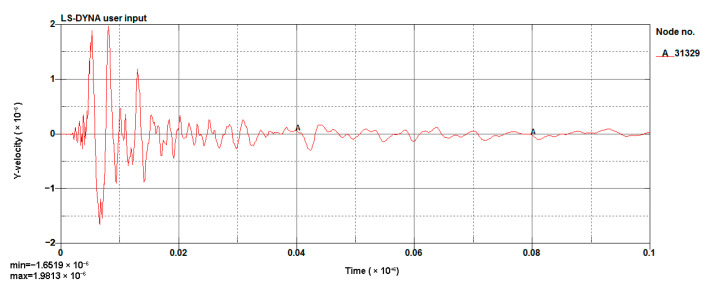
The waveform of scheme blasting of the lower pairs of holes for a 1m section (g-cm-µs).

Under the condition of using an ordinary detonator, it was found that the charge of scheme A in Section 5.1 was enough for the scheme of minimum velocity under such a condition. Thus, for the 1 m section, it was only necessary to verify whether the initiation sequence of scheme A met the requirement for velocity. Only the blasting vibration of the lower cutting hole was calculated and the scheme is shown as Figure 24. The result is shown in Figure 25.

The maximum vibration speed was 1.981 cm/s, which fulfilled the blasting design requirements. Because the maximum vibration velocity of the blasting had relative fluctuations of a certain value, it was not guaranteed that each blasting would meet the design requirements. 

**Figure 26:**
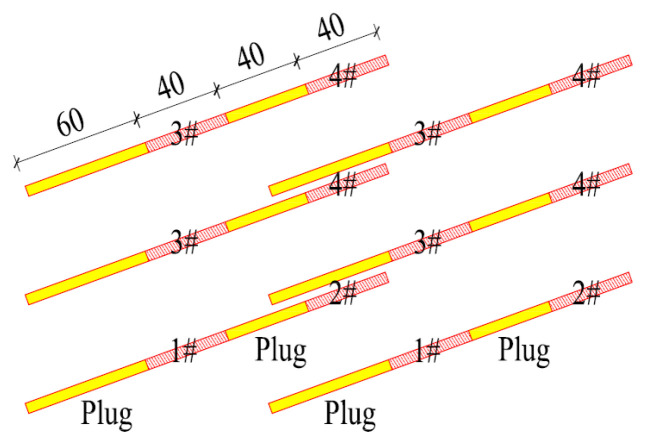
The footage charge structure (cm) for a 1.8 m section. The numbers in the figure represent the detonation sequence.

At this time, the wave interference vibration was reduced using high-precision electronic detonators, which could freely adjust the delay time. Two identical waves interfered were used to reduce the vibration. In order to generate the same waveform with double the magnitude, it was necessary to ensure the same charging structure and the same distance between the charging position and the surface buildings. The proposed wave interference vibration reduction scheme is shown in Figure 26. Its size was 1.8 m. The internal dynamite was 40 cm in length, the plug axial interval was 40 cm, the external dynamite was 40 cm, and the plugged section was 60 cm. The dynamite at point #1 was detonated first, followed by point #2, followed by point #3 and then point #4. The blasting time interval was 10 ms. This involved the detonation of the lower pairs of holes for point #1, followed by point #2, after a delay of 10 ms. The current blasting created free surface for the latter, so that the latter could generate a vibration of a similar size and mutual interference shock reduction. 

**Figure 27:**
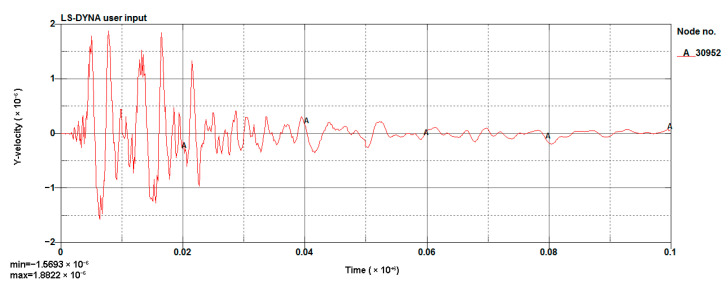
The waveform of the vibration reduction due to wave interference over a 1 m section (g-cm-µs).

The results are shown in Figure 27. The maximum vibration velocity was 1.882 cm/s, which met the vibration design requirements. The free surface was established through detonation of the previous blast, and the vibration generated during the current blast was reduced, resulting in interference. The current vibration established a free surface for the latter vibration and generated interference. Therefore, single-cycle footage could still be extended to longer than 1.8 m section.

**Figure 28:**
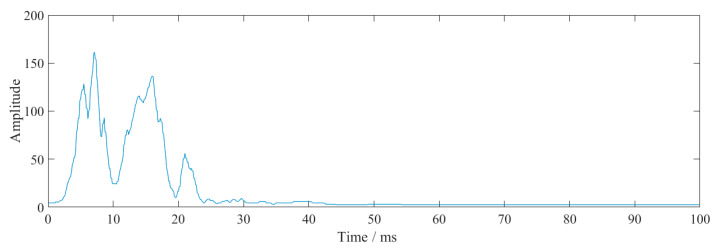
Instantaneous energy of the vibration reduction due to wave interference over a 1 m section.

Figure 28 shows the instantaneous energy obtained using HHT of the wave (Figure 27) through interference vibration reduction. There are two obvious main peaks in the figure, representing two blasts. The duration of energy in the superimposed waveform was relatively extended, which could potentially have negative impacts on the buildings as a result of energy accumulation.

According to the wave interference vibration reduction scheme, the vibration velocity fulfilled the necessary requirements. If the single detonation charge was small and the free surface could be continuously created, the single-cycle footage would be longer but the energy would be released continuously. Therefore, whether to use the interference vibration reduction method for vibration reduction construction depends on the energy tolerance limit of the surrounding structures and buildings.

The wave interference vibration reduction was effective and there was a theoretical optimal millisecond time. Because the conditions of the blasting object and the characteristics of the seismic wave propagation control varied greatly, the most reliable strategy was to determine the interference charging structure through field testing.

## 6. Conclusions

Based on the vibration monitoring signals measured above the palm face and surrounding buildings of the Xi’an-Chengdu High-Speed Railway Xiannvyan Tunnel, Hilbert−Huang Transform was used to obtain the instantaneous energy and marginal energy of blasting. Combined with finite element analysis to simulate the surface vibration characteristics of different cutting dynamites, effective vibration control schemes were proposed. Using waveform analysis and vibration simulation, the following conclusions were obtained.

The instantaneous energy spectrum obtained from HHT directly reflected the input energy of seismic wave to surface structures. The marginal spectrum reflected the accumulation of energy in a certain frequency section. The frequency of vibration wave analysis of event 2 was mainly between 0 and 50 Hz, and the vibration generated by cutting blasting was the largest.

The closer to the source, the larger the vibration frequency and velocity are. The larger the vibration velocity is, the lower the frequency is, and the greater the instantaneous energy is.

Three vibration reduction schemes were proposed based on the specific situation of the under crossing the Xiannvyan Tunnel for buildings: a 0.6 m footage scheme for simultaneously detonating cutting holes, a 0.8 m footage scheme for detonating cutting holes using a single segment charge, and a 1.8 m footage scheme using wave interference vibration reduction. The 0.6 m scheme was field-verified and could be used for the tunnel crossing under the building.

Ultra-shallow buried tunnels require comprehensive measures in order to control vibration. Such measures include shortening the blasting footage, reducing the charge of single-cycle dynamite, and manually excavating the free surface.

## Figures and Tables

**Table 1:** Blasting parameters.

Category of Holes	Number	Length (m)	Single Hole Charge (kg)	The Segments of Detonators (ms)
Cutting holes	4	0.6	0.6	1 (0 ms)
Auxiliary cutting holes	4	0.4	0.4	3 (50 ms)
	4	0.2	0.2	5 (100 ms)
	4	0.4	0.4	7 (200 ms)
	7	0.4	0.4	9 (310 ms)
Tunneling hole	13	0.4	0.4	11 (460 ms)
Periphery holes	36	0.2	0.2	13 (650 ms)
	28	0.2	0.2	15 (880 ms)

Note: dynamite specification: 40 mm, charge: 200 g/roll, length: 20 cm.

**Table 2:** Monitoring point characteristics and related parameters.

Monitoring Events	Monitoring Point	Epicentral Distance (m)	Source Depth (m)	Cutting Hole Charge (kg)	Vertical	
					Vibration Velocity (cm/s)	Vibration Frequency (Hz)
1	#1	75.00	11.90	4.2	1.206	86.957
	#2	0.00	11.79		7.548	28.571
2	#1	22.50	11.90	2.4	2.143	45.455
	#2	0.00	11.21		3.186	23.256

**Table 3:** Physical parameters of the rock model.

Density g/cm^3^	Elastic Modulus GPa	Poisson’s Ratio	Yield Strength MPa	Compressive Strength MPa	Internal Friction Angle°
2	25	0.23	75	80	32

**Table 4:** Soil material model.

Density g/cm^3^	Poisson’s Ratio	Cohesion KPa	Shear Modulus MPA	Internal Friction Angle°
1.86	0.35	61.2	52	33.25

**Table 5:** Model parameters and equation of state parameters of the dynamite materials.

Density g/cm^3^	Detonation Velocity cm/µs	Detonation Pressure MPa	*A* MPa	*B* MPa	*R*_1_	*R*_2_	*ω*	*E* MPa	Initial Specific Volume
1.23	0.42	0.097	2.144	1.82	1.23	0.42	0.097	2.144	1.82

**Table 6:** Blasting parameters of 0.6 m section.

Category of Holes	Number	Length (m)	Single Hole Charge (kg)	The Segments of Detonators (ms)
Cutting hole	6	0.2	0.2	1 (0 ms)

Note: dynamite specification: 40 mm, charge: 200 g/roll, length: 20 cm.

**Table 7:** Field blasting monitoring data.

Location of Monitoring Points	Vibration Velocity (cm/s)	Vibration Frequency (Hz)	Maximum Vibration Velocity Time (s)	Duration (s)
#2	1.852	36.036	0.00513	0.7

**Table 8:** Comparison of the field blasting data and simulation data.

Monitoring Points	Cutting Hole Charge (kg)	Vibration Velocity (cm/s)	Vibration Frequency (Hz)	Instantaneous Energy
#2 at event 1	4.2	7.584	28.571	2.5693 × 10^5^
#2 at event 2	2.4	3.186	23.256	0.7236 × 10^5^
#2 at event 3	1.2	1.852	36.036	0.2235 × 10^5^
0.6 m footage simulation	1.2	1.919	--	0.2588 × 10^5^

## Data Availability

Data can be provided when required.
